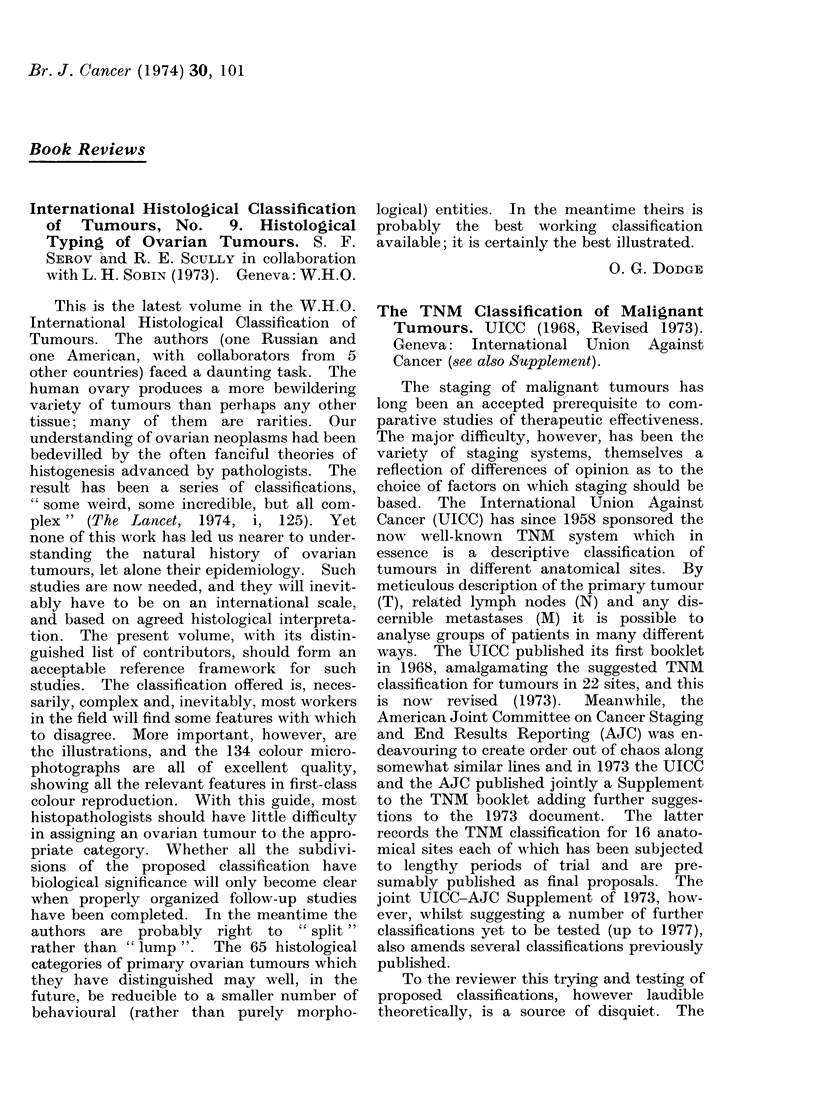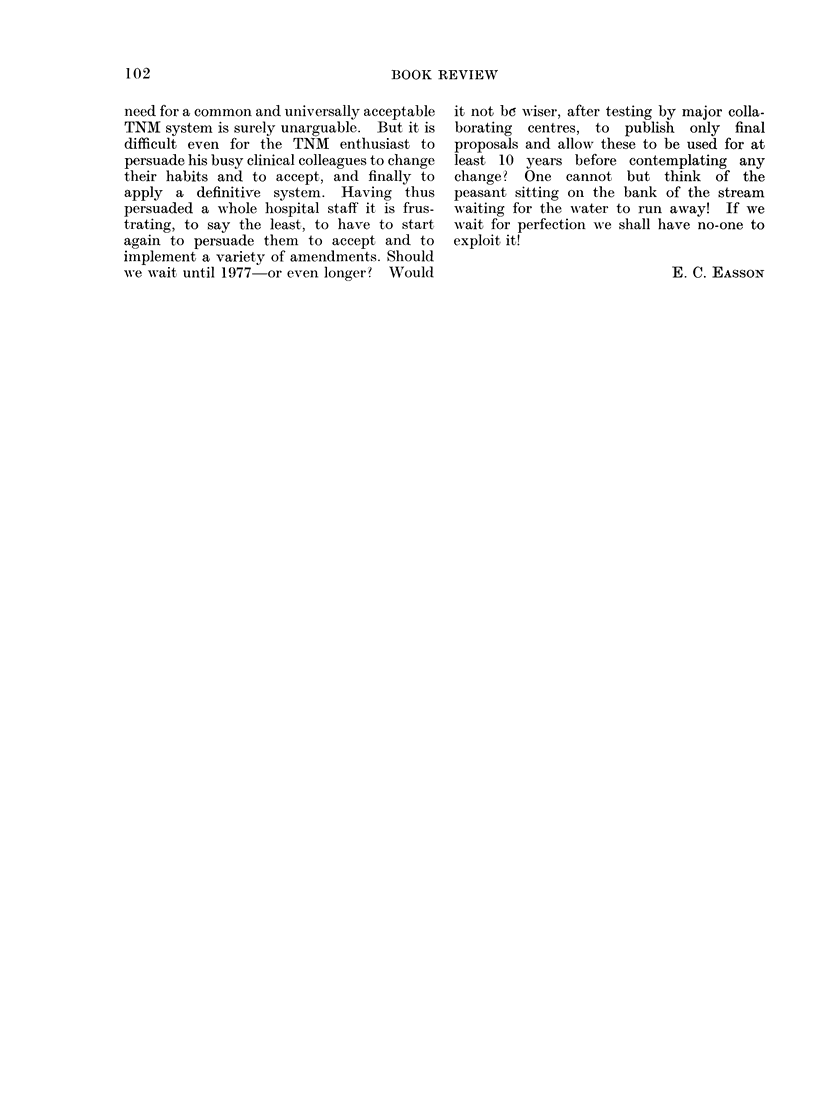# The TNM Classification of Malignant Tumours

**Published:** 1974-07

**Authors:** E. C. Easson


**The TNM Classification of Malignant Tumours.** UICC (1968, Revised 1973). Geneva: International Union Against Cancer (*see also Supplement*).

The staging of malignant tumours has long been an accepted prerequisite to comparative studies of therapeutic effectiveness. The major difficulty, however, has been the variety of staging systems, themselves a reflection of differences of opinion as to the choice of factors on which staging should be based. The International Union Against Cancer (UICC) has since 1958 sponsored the now well-known TNM system which in essence is a descriptive classification of tumours in different anatomical sites. By meticulous description of the primary tumour (T), related lymph nodes (N) and any discernible metastases (M) it is possible to analyse groups of patients in many different ways. The UICC published its first booklet in 1968, amalgamating the suggested TNM classification for tumours in 22 sites, and this is now revised (1973). Meanwhile, the American Joint Committee on Cancer Staging and End Results Reporting (AJC) was endeavouring to create order out of chaos along somewhat similar lines and in 1973 the UICC and the AJC published jointly a Supplement to the TNM booklet adding further suggestions to the 1973 document. The latter records the TNM classification for 16 anatomical sites each of which has been subjected to lengthy periods of trial and are presumably published as final proposals. The joint UICC–AJC Supplement of 1973, however, whilst suggesting a number of further classifications yet to be tested (up to 1977), also amends several classifications previously published.

To the reviewer this trying and testing of proposed classifications, however laudible theoretically, is a source of disquiet. The need for a common and universally acceptable TNM system is surely unarguable. But it is difficult even for the TNM enthusiast to persuade his busy clinical colleagues to change their habits and to accept, and finally to apply a definitive system. Having thus persuaded a whole hospital staff it is frustrating, to say the least, to have to start again to persuade them to accept and to implement a variety of amendments. Should we wait until 1977—or even longer? Would it not be wiser, after testing by major collaborating centres, to publish only final proposals and allow these to be used for at least 10 years before contemplating any change? One cannot but think of the peasant sitting on the bank of the stream waiting for the water to run away! If we wait for perfection we shall have no-one to exploit it!

E. C. Easson